# CABS-flex: server for fast simulation of protein structure fluctuations

**DOI:** 10.1093/nar/gkt332

**Published:** 2013-05-08

**Authors:** Michal Jamroz, Andrzej Kolinski, Sebastian Kmiecik

**Affiliations:** Faculty of Chemistry, University of Warsaw, Warsaw 02-093, Poland

## Abstract

The CABS-flex server (http://biocomp.chem.uw.edu.pl/CABSflex) implements CABS-model–based protocol for the fast simulations of near-native dynamics of globular proteins. In this application, the CABS model was shown to be a computationally efficient alternative to all-atom molecular dynamics—a classical simulation approach. The simulation method has been validated on a large set of molecular dynamics simulation data. Using a single input (user-provided file in PDB format), the CABS-flex server outputs an ensemble of protein models (in all-atom PDB format) reflecting the flexibility of the input structure, together with the accompanying analysis (residue mean-square-fluctuation profile and others). The ensemble of predicted models can be used in structure-based studies of protein functions and interactions.

## INTRODUCTION

Conformation flexibility is a key property of protein systems that is important for its biological function. Experimental knowledge of protein structural dynamics remains poor because of the limitations of experimental techniques. The majority of the known protein structure data are in the form of single static structures (solved by X-ray crystallography), not necessarily representing an active conformation. Recent experimental evidence supports the ‘conformational selection’ mechanism for molecular recognition (1), according to which all protein conformations pre-exist, and the binding partner selects the most favored conformation. A better understanding and more accurate prediction of proteins flexibility will have many practical applications in various areas, such as drug design, protein engineering and studies of molecular evolution (1).

The all-atom molecular dynamics (MD) simulations are considered the gold standard in simulating protein dynamics, but their computational cost is in many cases prohibitive for biologically relevant timescales. CABS, the well-established coarse-grained protein modeling tool, may generate consistent protein dynamics at highly reduced (three orders of magnitude) cost (2–6), although with some decrease of the resolution. The CABS-flex server follows the work of Jamroz *et al.* (2), where the authors demonstrated that the consensus view of protein near-native dynamics obtained from 10-ns MD simulations (all-atom, explicit water, for all protein metafolds using the four most popular force fields) is consistent with the CABS dynamics. The CABS-flex pipeline uses the CABS simulation protocol for near-native dynamics of globular proteins [for the development and optimization details see (2)]. The resulting trajectory is analyzed and clustered (and thus reduced) to a representative ensemble of protein models reflecting the flexibility of the input structure. An important attribute of protein models generated by the CABS model is that their spatial resolution (in Cα trace format) allows all-atom physically realistic models to be reconstructed [as shown previously in protein dynamics (3) and structure prediction (7,8) studies]. In the last step of the CABS-flex pipeline, the representative ensemble of predicted models is reconstructed to an all-atom representation followed by its visualization and conversion to data formats that are easy to analyze and use (e.g. as an input to docking methods or more exact simulation techniques).

As highlighted earlier in the text, there is a tremendous need for the structural description of protein flexibility in the form of 3D models and a lack of efficient tools for that purpose. For example, the major unsolved problem in protein–protein docking is the treatment of proteins with substantial backbone conformational change [as outlined in the review of the performance of protein docking techniques (9)]. Recent examples of web servers generating near-native conformational ensembles based on the input structure are NMSim (10) and RosettaBackrub (11) methods. The NMSim server implements simulation method based on normal-mode analysis, which is perhaps the most common approach to circumvent the computational cost associated with full dynamics simulations [implemented also in other servers (12,13)]. In contrast, the RosettaBackrub server uses a backrub sampling method (the backrub sampling involves internal backbone rotations about axes between Cα atoms) implemented in the Rosetta protein modeling program. Apart from a different prediction technique, the CABS-flex method distinguishes itself from the aforementioned tools by the kind and extent of validation studies. With regard to larger functionally relevant conformational changes (backbone movements larger than 1 Å), the RosettaBackrub and the NMSim benchmarks included, respectively, one (14) or a few cases (15) of experimentally observed conformational changes. The CABS-flex benchmark studies include theoretically simulated conformational changes (by the classical MD) of ∼400 proteins, as described later in the text.

## MATERIALS AND METHODS

### Input structure file

The only data required as an input are a protein structure file (given as a PDB code or uploaded by a user). The input structure file must be provided in PDB format (http://www.wwpdb.org/docs.html). Only single and continuous (without breaks) protein chains (up to 400 standard amino acids in length) are accepted. Non-standard amino acids are not accepted. Each residue must have a complete set of backbone atoms (N, Cα, C and O); side chain atoms may be missing. If multiple conformations for any single residue are provided in the PDB file, the CABS-flex uses only the first one. Heteroatoms (e.g. water or ligands) are not considered in the simulations. It is possible to upload PDB files containing alternative protein structures (such as determined by nuclear magnetic resonance methods), but then only the first model in the PDB file is used as an input.

### The CABS modeling protocol and its validation

**Figure 1.**
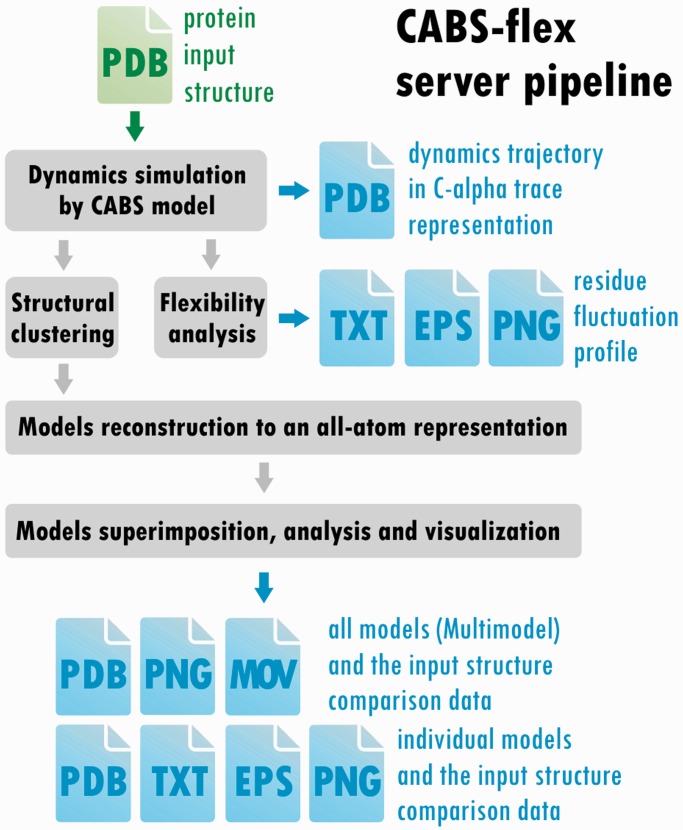
The CABS-flex server pipeline. Output files (marked in blue) are generated in the following, ready-to-use, file formats: PDB, Protein Data Bank (for entire dynamics trajectory in Cα trace representation as well as the trajectory representatives in all-atom resolution); TXT, text; PNG, Portable Network Graphics (online displayed graphics); EPS, encapsulated PostScript graphics; MOV, movie (online displayed, and to be download in OGV or MP4—depending on web browser—file formats).

As shown in the scheme presented in Figure 1, the input structure is used as a starting point for the CABS simulation procedure for near-native dynamics (2). Apart from the input structure coordinates, the secondary structure data automatically assessed by the DSSP method (16) (and reduced to the helix/β/coil definitions only—other definitions are treated as a coil region) are used by the CABS model.

The CABS model [described in details elsewhere (17)] uses high-resolution coarse-grained representation of a protein chain, in which a single protein residue is represented by up to four atoms (the Cα and Cβ atoms and two virtual pseudo-atoms: the center of mass of a side chain and the center of the Cα−Cα virtual bond). The CABS force field includes knowledge-based statistical potentials (sequence-dependent short-range conformational preferences, context-dependent potential of pairwise interactions of side chains and a model of the main chain hydrogen bonds) accounting for the solvent effect in an implicit fashion. The CABS dynamics are simulated by a random series of small local moves (controlled by Monte Carlo scheme) whose long-term evolution describes the protein dynamics well (2–6).

The CABS simulation results in the 2000-snapshot trajectory in Cα trace representation (available for download in the PDB format). Based on the generated trajectory, a residue fluctuation profile (mean-square-fluctuation) is calculated as

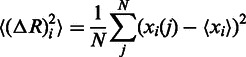

where <> denotes the average over a whole trajectory, and *x* is the position of particle *i* in the frame *j*.

The residue fluctuation profile (available for download in txt, eps and png file formats, Figure 1) shows relative propensities of protein residues to deviate from an average dynamics (trajectory) structure. The residue fluctuation values are also included into the final PDB output models (the temperature factor column, 61–66 columns in the PDB file, is replaced with the fluctuation values that can be visualized as colors using standard molecular visualization software).

**Figure 2.**
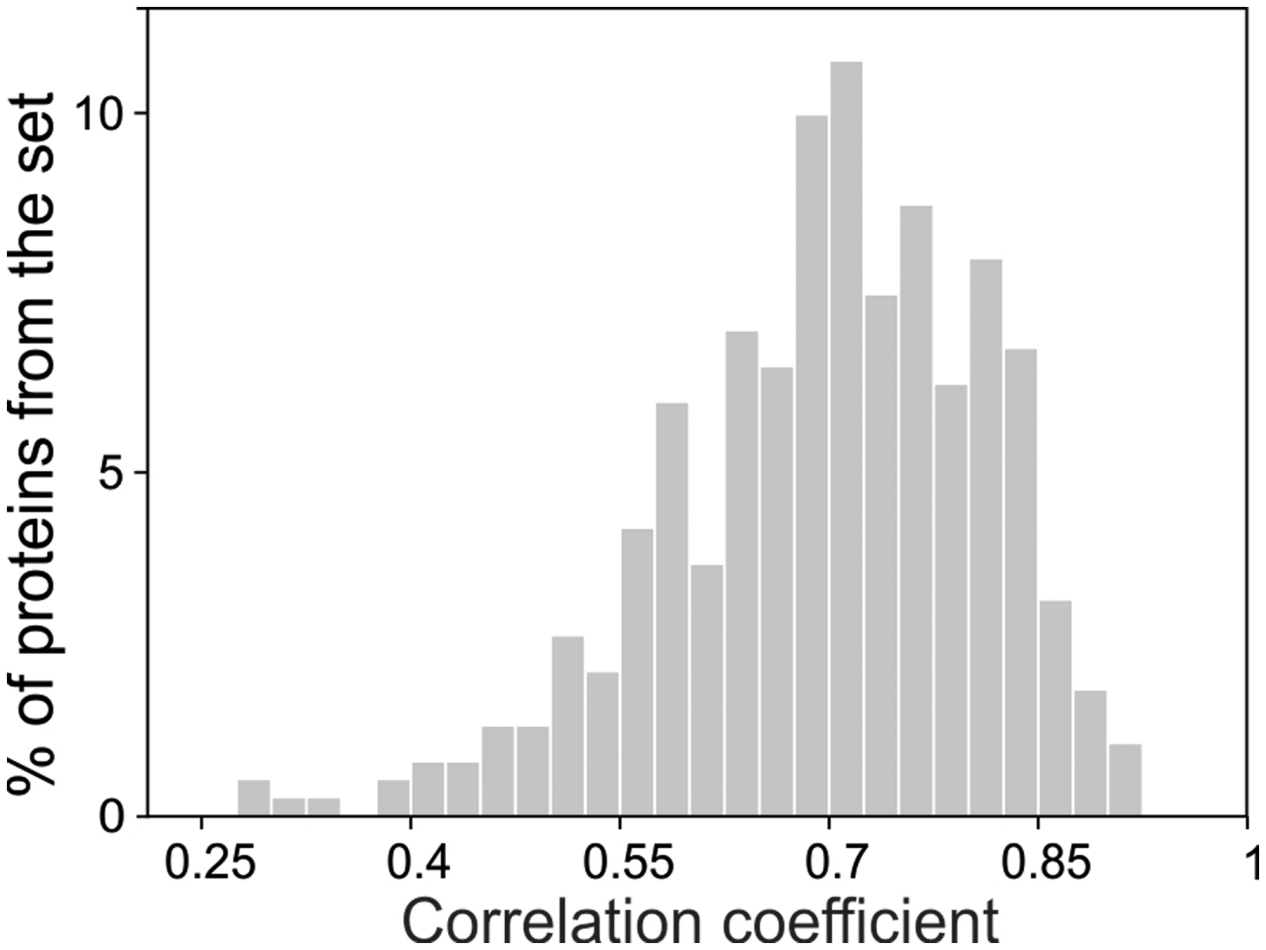
Spearman’s correlation coefficient between MD and CABS-flex fluctuation profiles for the test set of 393 proteins.

The CABS simulation procedure was parameterized and validated using the microMoDEL subset (18) of the MoDEL database (19). The microMoDEL subset consisted of the MD simulations (all-atom, explicit water), using the four most popular force-fields (Amber, Gromos, OPLS and Charmm) for each of 22 proteins representing different metafolds. The average Spearman’s correlation coefficient for the residue fluctuation profiles between CABS and MD, from the 2-fold cross-validation test on the microMoDEL set was shown to be 0.7 [this consistency measure as well as other dynamics metrics seemed to be close to those found when different MD force fields are compared (2)]. This level of prediction is better than analogical predictions recently achieved by other methods: support vector regression and Gaussian network model [respectively, 0.67 and 0.64, as presented previously in Jamroz *et al.* (20)]. In addition, we validated the method on a parameterization-independent set of proteins having 10-ns MD trajectories deposited in the MoDEL database (19). The protein test set consisted of 393 non-redundant proteins (<30% of sequence identity), up to 400 residues in length. The results confirmed the earlier validation test and showed the average Spearman’s correlation coefficient for the residue fluctuation profiles between CABS and MD equals 0.70 (the histogram of the correlation coefficients obtained for the test set is shown in Figure 2). The detailed benchmark results are presented within the CABS-flex online documentation under the following link: (http://biocomp.chem.uw.edu.pl/CABSflex/benchmarks.php).

### Post-processing and data analysis

**Figure 3.**
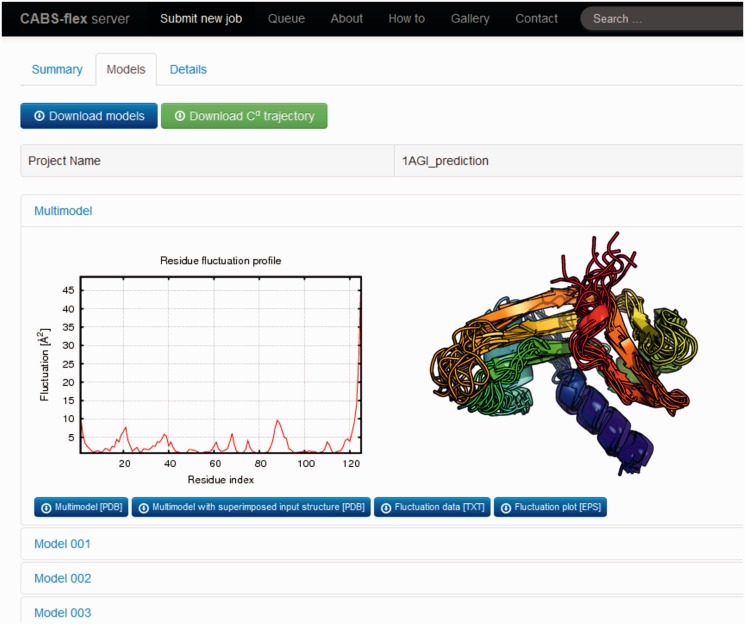
Screenshot of the CABS-flex server example results (top of the models tab) for 1AGI pdb file is presented (showing in the foreground the residue fluctuation profile and the picture of predicted ensemble).

Three consecutive steps follow the CABS simulation procedure: (i) structural clustering, (ii) models reconstruction to an all-atom representation and (iii) models superimposition, analysis and visualization (Figure 1). All these tasks are performed in the CABS-flex pipeline by well-established and extensively tested methods (typical for multi-scale protein modeling procedures) or general purpose scientific software. The structural clustering is done by a classical K-means method (for each prediction, the detailed clustering results are given under the details tab). After clustering is done, each cluster representative is chosen (always the model whose average dissimilarity to all models in a cluster is minimal). Predicted protein models, presented in the models tab (Figure 3), are each cluster representatives (the clusters and the corresponding models are marked by the same numbers, e.g. Model 1 represents Cluster 1). The clusters are numbered/ranked according to cluster density values, from the most dense (numbered as the first) to the least dense one.

The next post-processing step, i.e. models reconstruction, is realized by the BBQ (21) (Backbone Building from Quadrilaterals algorithm for the reconstruction of backbone atoms from Cα trace) and subsequently by the ModRefiner method (22) (responsible for the reconstruction of side chain atoms and final models optimization). As recently presented (22), in comparison with other state-of-the art programs, the ModRefiner shows improvements in both global and local structures, which have more accurate side chain positions, better hydrogen-bonding networks and fewer atomic overlaps.

The final post-processing step involves multiple superimposition tasks performed by the Theseus software package (23). The Theseus simultaneously superimposes multiple protein structures and finds the optimal solution to the superposition problem using the method of maximum likelihood. By downweighting variable regions of the superposition and by correcting for correlations among atoms, the maximum likelihood superpositioning method brings much more accurate results than conventional methods using least-squares criteria (23). The following superimposition tasks are automatically performed: on the ensemble of all predicted models, on the ensemble of all predicted models and the input structure and between the input structure and each predicted model. All the superimposition data (in the form of pdb, txt or eps file formats) are available for download, or visualized (as png graphics), under the models tab and its subtabs (Multimodel, Model 1, Model 2 and others, for the example screenshot see Figure 3). Additionally, a movie is automatically generated by creating frames from pictures of the predicted models in different rotational states, and it is made available under the summary tab [example movies, for proteins of different size and architecture (pdb codes: 1ass, 1hui, 1k40, 1mhn and 1qhd), are available in the Supplementary Data].

For the generation of online presented plot pictures and eps plot files, Gnuplot 4.5: an interactive plotting program is used. For the generation of online presented pictures, and movie showing an ensemble of predicted models in the cartoon representation, Open Source PyMOL visualization software is used.

### Documentation

The documentation of the CABS-flex is available online, and it can be accessed using the links in the menu at the top of every server page. It contains a description of the method, a tutorial, benchmark data and a gallery of predicted protein ensembles. Additionally, the web interface provides short help notes for download buttons or output graphics (to display a help note drag the cursor over a picture, or a particular download button). The online documentation is updated on a regular basis according to users’ needs or the method improvements.

### Availability

The CABS-flex server is free and open to all users, and there is no login requirement. After clicking submit button (preceded by filling the input—project name and pdb file—data), a web link to the results is provided, which the user can bookmark and access at a later time. Web links to the submitted jobs are displayed on a queue page, unless the option ‘Do not show my job on the queue page’ (available from the submit page) is marked.

## SERVER ARCHITECTURE AND PERFORMANCE

The CABS-flex server is equipped with a web interface, written in html + php, which provides a convenient framework for the pipeline control and the presentation of the output data. User-provided data are validated by PHP script (correctness of model and so forth) and added to MySQL database (‘pending’ status). A cron daemon script checks new records in the database and (if any exist) sends the job to the queue (‘in_queue’ status). If the server has free resources, the job starts (‘running’ status) invoking bash and python scripts using database data. Typically, the computations take between 2 and 3 h. As soon as the CABS-flex pipeline (described in Figure 1) is complete, the job status is set to ‘done’. The job status, as well as a progress bar (showing approximate job progress), is constantly displayed under a unique job link.

The server is equipped with standard SGE 6.1 queue manager with the maximum number of parallel running tasks set to eight (of 12). Every 2 min the daemon checks whether there are any new tasks, and if they exist, it adds them to the queue manager. The runtime of a simulation depends mostly on protein chain length and varies between 2 and 4 h (the approximate finish time is given under the job link).

## SUPPLEMENTARY DATA

Supplementary Data are available at NAR Online: Supplementary Movies 1–5.

## FUNDING

Foundation for Polish Science MPD programme and TEAM project [TEAM/2011-7/6] co-financed by the EU European Regional Development Fund operated within the Innovative Economy Operational Program; Polish National Science Centre [NN301071140]; Polish Ministry of Science and Higher Education [IP2011 024371]. Funding for open access charge: Polish National Science Centre [NN301071140].

*Conflict of interest statement.* None declared.
